# Transcranial alternating current stimulation ameliorates emotional attention through neural oscillations modulation

**DOI:** 10.1007/s11571-022-09880-5

**Published:** 2022-11-12

**Authors:** Shuang Liu, Yuchen He, Dongyue Guo, Xiaoya Liu, Xinyu Hao, Pengchong Hu, Dong Ming

**Affiliations:** 1 Academy of Medical Engineering and Translational Medicine, Tianjin University, 300072 Tianjin, China; 2 Department of Biomedical Engineering, College of Precision Instruments and Optoelectronics Engineering, Tianjin University, 300072 Tianjin, China; 3 Tianjin International Joint Research Center for Neural Engineering, 300072 Tianjin, China

**Keywords:** Transcranial Alternating Current Stimulation, Emotional attention, Alpha oscillation, Individual alpha frequency, Dorsolateral prefrontal cortex

## Abstract

**Background:**

Numerous clinical reports have suggested that psychopathy like schizophrenia, anxiety and depression is accompanied by early attentional abnormalities in emotional processing. Recently, the efficacy of transcranial alternating current stimulation (tACS) in changing emotional functioning has been repeatedly observed and demonstrated a causal relationship between endogenous oscillations and emotional processing.

**Aims:**

Up to now, tACS effects on emotional attention have not yet been tested. To assess such ability, we delivered active-tACS at individual alpha frequency (IAF), 10 Hz or sham-tACS for 7 consecutive days in the bilaterally dorsolateral prefrontal cortex (dlPFC) to totally 79 healthy participants.

**Results:**

IAF-tACS group showed significant alpha entrainment at-rest, especially in open state around stimulation area and showed an obvious advantage compared to 10 Hz-tACS. Event-related potential revealed a significant larger P200 amplitude after active-tACS and IAF group showed wider range of emotions than 10 Hz-tACS, indicating the attentional improvement in facial emotion processing. A notable positive correlation between alpha power and P200 amplitude provided an electrophysiological interpretation regarding the role of tACS in emotional attention modulation instead of somatosensory effects.

**Conclusion:**

These results support a seminal outcome for the effect of IAF-tACS on emotional attention modulation, demonstrating a feasible and individual-specific therapy for neuropsychiatric disorders related to emotion processing, especially regarding oscillatory disturbances.

## Highlights

Proposing a non-invasive treatment on emotional attention–long-term tACS treatment.

Exploring the correlation between neural oscillations and the performance of emotion recognition tasks and demonstrating the emotional attention modulation by leveraging neural entrainment of weak alternating electric fields.

Demonstrating the entrainment effect of individual tACS strategy in the individual frequency band for each subject, providing a feasible and individual therapy in neuropsychiatric disorders with abnormal rhythms.

## Introduction

Emotion plays an important role in daily life. Recently, emotion processing has been under intensive focus in the field of human neuroscience(Dolcos et al. 2020). Clinical research has indicated that the numbness to emotional information for psychopathy may be explained by early attentional abnormalities(Connell et al. 2019; Martínez et al. 2019). As the initial stages of emotion processing, early attention to emotional cues always occurs prior to emotional information extraction and contributes to the subsequent emotional recognition and evaluation processing. However, the mechanisms underlying early attentional stage for emotion processing remain unclear, which necessitates preclinical research with healthy subjects to access effective treatments and facilitate clinical applications.

As one of the classical approaches in emotion processing research, facial processing marked by rapid attentional processing caused great concern due to distinct responsiveness of early components to faces(Jessen and Grossmann 2020). Given its excellent temporal resolution and sensibility to specific psychological processes, the time course of facial emotion processing has been mostly investigated using event-related potentials (ERPs). In particular, early ERP components (100–300ms) such as N100 and P200 usually relate to attentional processes and are considered as indices of early attention to emotional stimuli. While later components (> 300ms) such as P300 have been thought to reflect more elaborative and top-down emotional information processing, including face feature encoding and emotional evaluation(Fields and Kuperberg 2012). Therefore, exploring the relationship between early components to facial stimuli and initial psychological processes in emotional tasks is of great importance.

Apart from dynamic course, spectrum analysis also contributes to uncovering brain mechanisms(Bocchio et al. 2017). Electrophysiological studies have demonstrated a possible association between emotion processing and brain oscillations(Jacobs et al. 2007). Alterations or disturbances in particular oscillations can be linked to psychiatric disorders(Sohal 2012; Kang et al. 2018). In some cases, brain oscillations are amenable to external intervention by neuromodulation techniques(Yavari et al. 2018). As a non-invasive stimulation paradigm, transcranial alternating current stimulation(tACS) has potential to entrain endogenous cortical oscillations via employing weak sinusoidal electric current to the scalp(Krause et al. 2019; Johnson et al. 2020). Much of interest in tACS stems from its frequency-specific manner, which offers the possibility to facilitate causal relationship between targeting oscillation and characterized cognitive processing(Hanslmayr et al. 2019; Krause et al. 2019). Considering the neural modulation of tACS and the brain mechanism under emotion attention process, its performance on emotional attention via neural oscillations modulation becomes an emerging issue.

Two factors count in addressing the hypothesis above: frequency band and brain regions. Alpha band (8–13 Hz), known initially as “idling rhythm”, operates through power alternation and sustains higher intrinsic cortical activity(Zheng et al. 2019). Numerous empirical evidences links alpha oscillations with activities of emotional and attentional systems during affective tasks, particularly in clinical studies(Eidelman-Rothman et al. 2016). Depression-related alpha abnormalities are mostly centered at frontal and posterior alpha asymmetry and inconsistent alpha power alternations(Harmon-Jones et al. 2008). Studies in anxiety, and post-traumatic stress disorders also underline the relationship between alpha abnormalities and psychopathology(Crost et al. 2008; Imperatori et al. 2014). In general, studies assessing alpha activity often focus on fixed or individualized alpha bands, and the latter has been suggested to be more advantageous, since it takes into account an individual’s alpha characteristics(Bazanova and Vernon 2014). Altogether, these observations indicated the role of alpha functionality in attentional and affective processing.

As for the target brain region, evidence indicated that the prefrontal cortex is involved in emotion-related functions. As part of the dorsal system, the dorsolateral prefrontal cortex (dlPFC) plays central role in cognitive regulation and executive function of emotion(Dixon et al. 2017; Raschle et al. 2019). Neuroimaging and electrophysiological studies have provided great insight into the link between dlPFC abnormalities and psychiatric disorders(Liston et al. 2014). Although explicit correlation between dlPFC deficit and emotional function has been well-established, it remains partially unclear how the dlPFC activity affects emotion processing.

Given the emotional function of the dlPFC and attention-affective role of alpha rhythm, alpha-tACS over dlPFC can be considered as an intermediate to explore their interactive connections. It also promotes the investigation of early attentional stages during emotion processing, which is the pioneer preclinical research in the field. Herein, a single-blind and sham-controlled experiment was conducted to validate tACS effect of on promising emotional attention and compare the effectiveness of individual-tACS with tACS at fixed frequency, aiming at providing a potential regulation approach regarding emotion processing.

## Methods

### Participants

A total of 79 right-handed healthy participants (38 females, mean age = 23.64 years, age range = 20 to 27 years) participated in the study. All of the participants, with normal or corrected to normal vision, gave written informed consent and reported no history of neurological or psychiatric disorders or prior transcranial electrical stimulation experience. Prior to the experiment, participants were instructed to complete mood questionnaires using the State-Trait Anxiety Inventory (STAI; Spielberger et al., 1968) and Difficulties in Emotion Regulation Scale (DERS; Gratz & Roemer, 2004) to access self-reported emotion regulation functioning(Barnes et al. 2002; Bardeen et al. 2012).

### Experimental design

The experiment involved a randomized, single-blind, sham-controlled design and was performed in an electrically shielded and sound-attenuated cabin. All of the procedures were conducted with approval from the Institutional Review Board at Tianjin University and ethics committee of Tianjin University Tianjin Hospital. The study was registered as an interventional clinical trial with the number of NCT04551118.

**Fig. 1:**
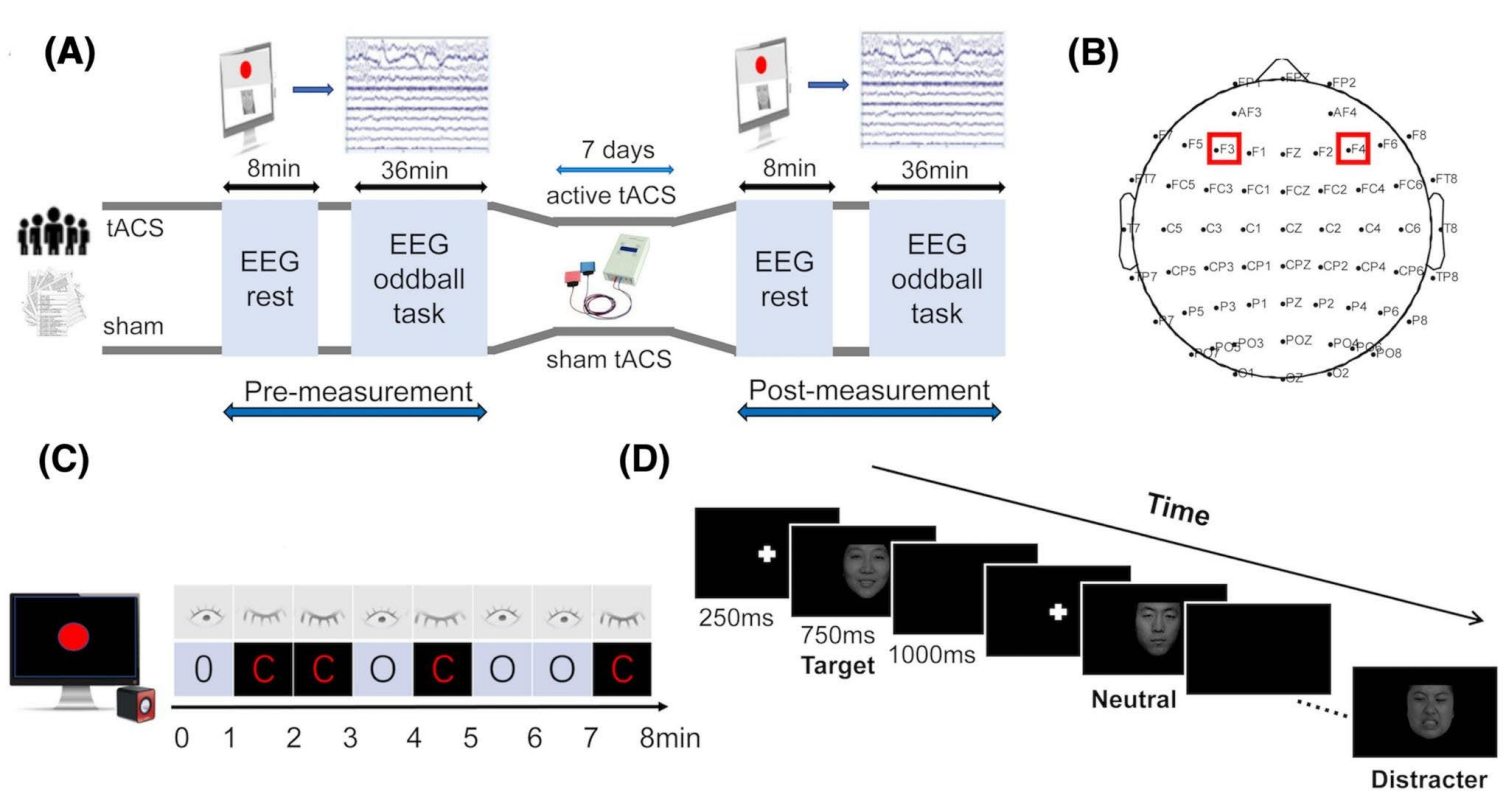
Experimental paradigm. (A) Experimental procedure. (B) EEG and tACS electrode placement on the scalp. (C) Illustration of 8 min resting task with eyes close (C) and open (O). The periods consisted of 8 one-minute intervals of rest, ordered in OCCOCOOC. Participants need to concentrate on the red circle on the screen in the open intervals. After the last interval, participants would hear a long “beep” sound with the red circle turned blue, indicating the end of the procedure. (D) Illustration of facial emotion identification task. Shown is a possible order of pictures in the positive condition, with each stimulus presented in the center of the screen for 1 s, including 250 ms for fixation cross and 750 ms for face stimulus, followed by a blank screen for 1000 ms. The task was preceded by a short practice period. In each block, participants kept their eyes fixated on the screen and responded as quickly as probable to the target stimuli by pressing the space bar and ignoring distracter stimuli as well frequently presented standard stimuli. To avoid the leakage of identifying information of people, the actual facial stimuli were replaced with graphics in the flowchart

The procedure was clearly explained to all participants beforehand. The entire procedure required 7 days (Fig. 1 A). EEG recording was executed repeatedly on day 1 (before stimulation) and day 7 (after stimulation). During EEG recording, each participant underwent two tasks, including 8 min of resting EEG with eyes open and close task and facial emotion identification task. In each task, the participants were asked to relax, minimize their body movement to reduce the appearance of relevant artifacts in the EEG recordings, and concentrate.

### tACS

tACS was administered through a battery-operated stimulator (DC Stimulator, Neuroconn, Germany) and applied in sinus pattern with the initial phase of 0, which is deemed as the optimal phase difference for task performance(Kanai et al. 2008). Two round rubber electrodes (2.5 cm diameter) in saline-soaked sponges were placed bilaterally over F3/F4 of the international 10–20 system (Fig. 1B). The impedance was kept below 10 KΩ of stimulation duration. Stimulation intensity was 1.5 mA (peak-to-peak), below individual phosphene and discomfort threshold. All participants were randomized to 3 groups, 1 group for sham stimulation and 2 groups for active tACS at individual alpha frequency (IAF) or 10 Hz. IAF was selected for each participant on day 1, given that alpha frequency varies among age, memory and genetic background. We defined IAF as the prominent alpha peak of the eyes-open spectrum at stimulation site, which ranging from 8 to 11 Hz across participants.

In order to distinguish tACS effect between neural entrainment and somatosensory induced by current shunting, we conducted an additional group of 22 participants using DC stimulation from the same population and recorded their EEG during facial emotion identification task. Participants in each group received stimulation for 7 consecutive days for a duration of 20 min each day at about the same time (± 2 h). More details for tACS setting can be found in the *Appendix*. Regardless of the authenticity of the stimulation, all participants were required to remain relaxed during the stimulation process.

### Tasks

For the resting task, EEG acquisition started with 8 min of resting EEG. During recording, participants were instructed to remain still and relaxed with their eyes open (O) and closed (C) in two alternating orders following the voice prompts (Fig. 1 C), with the goal of averaging the difference between open and closed signals(Adolph and Margraf 2017). For the facial emotion identification task, a well-established visual oddball paradigm was used to access neurophysiologic effects of incidental emotion processing and retrieve ERP components (Fig. 1D). Participants were instructed to complete six blocks made of three positive and three negative conditions. The block order was random for each participant. Each block lasted for 6 min and consisted of a pseudorandom series of 180 pictures with positive, negative and neutral faces from the Chinese Affective Picture System (CFAPS). Within each block, facial stimuli were presented with overall proportions of 70% standard stimuli, 15% targets and 15% distracters. Facial stimuli with emotional valence opposite to the targets were used as distracters (e.g., positive distracters in the block with negative targets), and neutral faces were used as standard stimuli.

### EEG processing and statistical analysis

Raw EEG data was acquired by a SynAmps2 amplifier using a 64-channel EEG acquisition system (NeuroScan, Germany) sampled at 1,000 Hz. The right aural tip was served as reference and AFz was served as ground. The analysis included preprocessing, power spectral estimation, and ERP analysis.

For spectral analysis, the resting EEGs were separated according to the eyes-open and eyes-closed state, then epoched into 5 s segments, after which power spectrum was estimated with a 1024-point fast Fourier transform (FFT) using Welch’s method (0.5 Hz resolution). The resulting spectrum was subsequently averaged across epochs. To determine alpha changes between day 1 and day 7, averaged spectrum and alpha power at electrode level were accessed for each subject. The full and individual alpha power was obtained by the integral of power spectrum values from 8 to 13 Hz and around the IAF (IAF − 1 Hz ≤ IAF ≤ IAF + 1 Hz) respectively, then spatially-normalized across electrodes to eliminate the individual alpha variability(Ahn et al. 2019). A 2-way repeated measures ANOVA (analysis of variance, Greenhouse-Geisser corrected) with within-group factors of time (pre- and post-stimulation) and between-group factor group (active and sham group) was conducted with normalized alpha power. Before statistical analysis, extreme data were eliminated from dataset. Post hoc analysis was performed using Bonferroni-corrected t-tests with the significance level at p < 0.05.

Then, ERP elicited by positive, negative, and neutral faces were analyzed using the signals recorded during facial emotion identification task. In this study, N100 (60−120 ms), P200 (120−200 ms) and P300 (350−550 ms) components were measured by peak value (baseline to peak of each component) and latencies (from stimulus onset to the peak) at Pz electrode based on grand average ERP activity and previous studies(Junghöfer et al. 2001; Luo et al. 2010; Alzueta et al. 2019), which reflects the degree to which component-related psychological process are engaged and the time point of completion for the psychological operation respectively(Campanella et al. 2002). The specific method for preprocessing and ERP analysis can be found in the *Appendix*. Then, we computed peak amplitudes and latency differences for each component. To assess tACS effects on ERP modulation, a 3-way repeated measures ANOVA with two within-group factors of time (pre- and post-stimulation), condition (Target, Neutral and Distracter) and one between-group factor group (active and sham group) was conducted.

Pearson’s correlation test was used to assess possible correlations in factor analysis for neurophysiological mechanism of ERP modulation of tACS. Statistical analyses were performed using SPSS (SPSS Statistics, version 25; IBM Corp).

## Results

### Alpha spectrum

To explore the effects of tACS on neuronal entrainment, changes in resting-state power spectrum in fixed and individual frequency band were assessed. The deviation in spectrum and alpha power was defined as relative changes calculated by the post- minus the pre-stimulation data. ANOVA for spatially-normalized full alpha power of frontal lobe showed a significant factor time in 8 min resting data (F_1,72_ = 5.89, p = 0.18). The factor group (F_2,72_ = 0.65, p = 0.52) were not significant. While their interaction was significant (F_2,72_ = 3.59, p = 0.018). A post hoc analysis revealed that after IAF-tACS, alpha activity around stimulation site was significantly amplified compared to 10 Hz and sham group (p = 0.001).

**Fig. 2:**
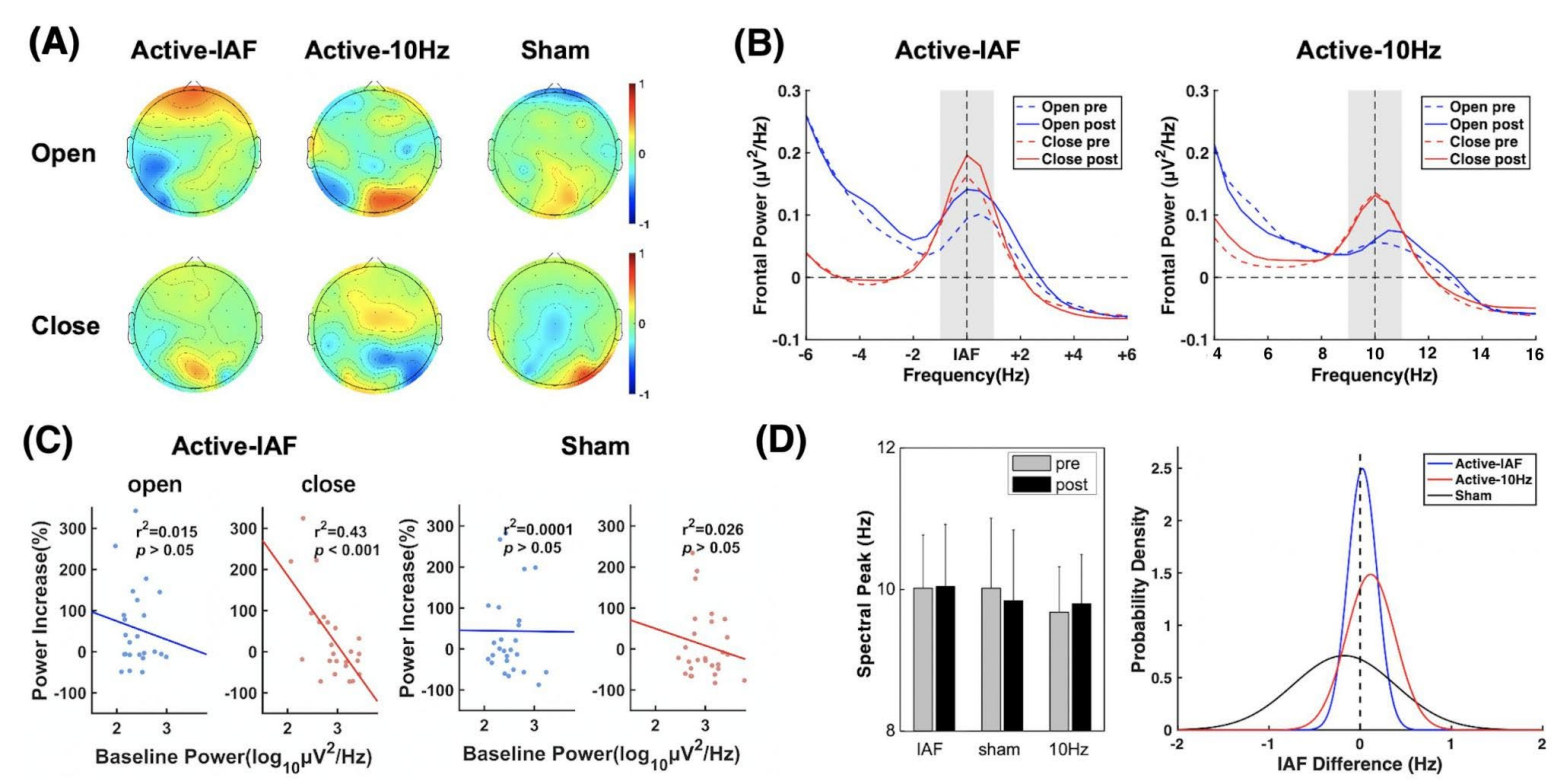
Alpha activity changes after 7 day tACS in open and close states. a. (A) The topography of the individual alpha power change after stimulation for 3 groups. The top row shows open power change and the bottom row shows close power change. b. (B) Mean power spectra of frontal lobe across subjects for active IAF-tACS (left) and 10 Hz-tACS (right) groups. Gray shade represents the individual alpha band. Open pre = frontal power of open state before stimulation; Open post = frontal power of open state after stimulation; Close pre = frontal power of close state before stimulation; Close post = frontal power of close state after stimulation. c. (C) Linear correlation analysis of the absolute alpha power on day 1 and the alpha power increases active IAF-tACS (left) and sham groups. Each dot depicts one subject. Significance of Pearson correlation (p) and R-square (r2) are marked in the scatterplots. d. (D) Spectral peak analysis in alpha band of resting EEG for 3 groups. The histogram shows the mean spectral peak pre- and post-stimulation (left) and the probability density shows the distribution of spectral peak difference (right). Pre = peak frequency before stimulation; post = peak frequency after stimulation

To further verify specific alpha changes after stimulation at rest, changes in individual alpha power in open and close states were obtained for three groups. As shown in Fig. 2 A, an obvious frontal alpha promotion was found after IAF-tACS in open state, compared to 10 Hz tACS and sham group. However, there was slight alternation in close state of three groups. Further, we compared alpha spectrum changes in frontal area for two active groups. The frequency axis was aligned to IAF of each individual in order to explore individual alpha changes, as shown in Fig. 2B. The results showed that individual alpha activity was amplified after IAF-tACS, which is more obvious in open state. However, the 10 Hz group does not show the superiority of the frontal alpha change. Subsequent ANOVA for frontal alpha power in open state showed that the interaction of time and group was significant (F_2,73_ = 3.49, p = 0.036). While the factor time (F_1,73_ = 0.36, p = 0.55) and group (F_2,73_ = 0.30, p = 0.74) were not significant. The significant individual alpha enhancement was found only in IAF-tACS group from post hoc analysis (p = 0.017). Besides, alpha entrainment was not found in adjacent frequency bands (IAF − 3 Hz ≤ IAF ≤ IAF − 1 Hz and IAF + 1 Hz ≤ IAF ≤ IAF + 3 Hz, all p > 0.05), exhibiting the spatial and frequency specificity of tACS entrainment. Considering the IAF stems from eye-open data, the similar phenomenon was not found in close state (all p > 0.05).

In addition, a significant negative correlation in the close state was found between the log-transformed absolute alpha power on day 1 and the relative increase in alpha power after IAF-tACS (r^2^ = 0.43, p = 0.00048, Fig. 2 C). The similar phenomenon was not observed in the open state (r^2^ = 0.015, p = 0.57) or sham group (r^2^ = 0.026, p = 0.42). This finding indicated that was limited by initial alpha power in the close state. That is, the IAF-induced alpha enhancement in 8 min resting data power was due to individual alternation in open state.

Figure 2D showed the tACS-effect of IAF. The peak frequency of the alpha band in IAF-tACS group was almost unshifted after stimulation compared to the sham group and exhibited a slight change for each subject, suggesting a “locking effect” of neural activity in the stimulation band. While the spectrum peak in fixed stimulation group was close to 10 Hz after tACS. Besides, there were no significant IAF differences found between groups.

### ERPs

**Fig. 3:**
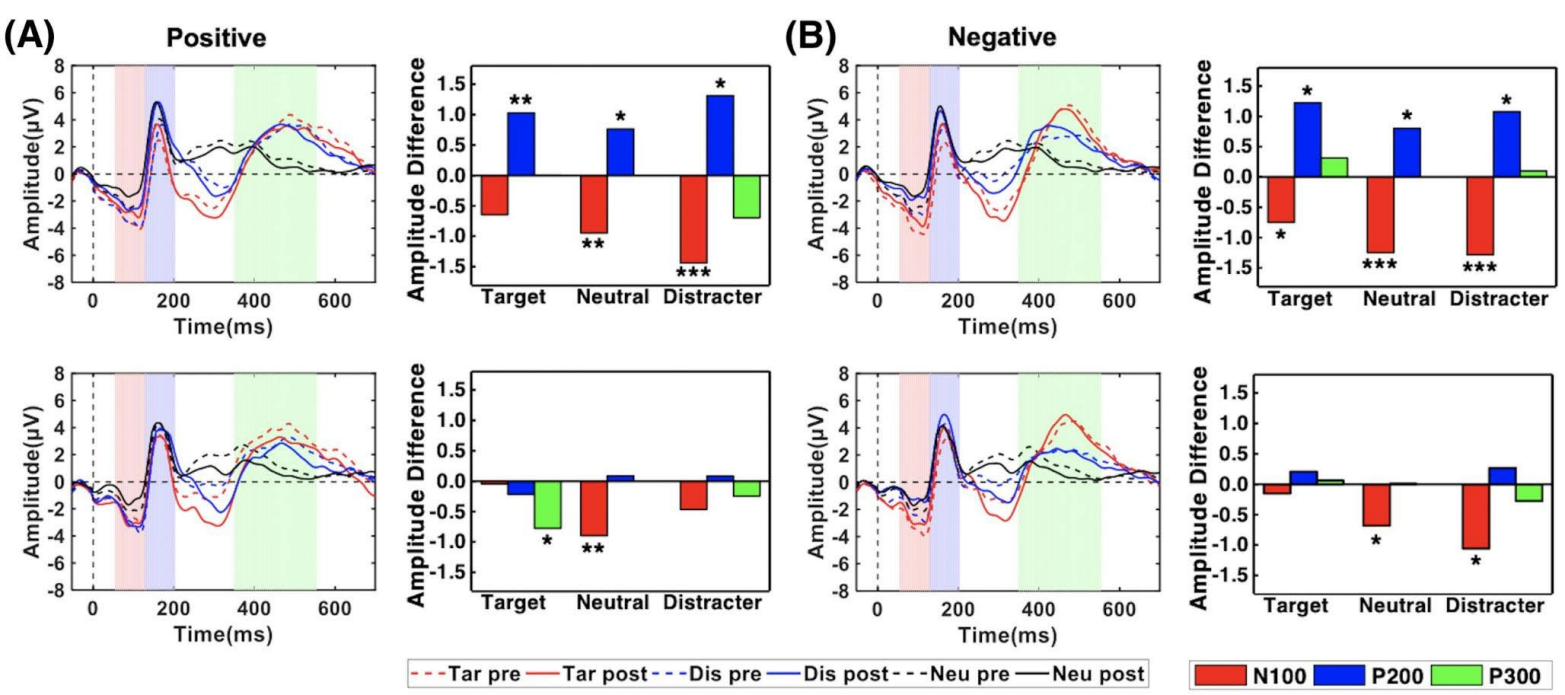
ERPs generated by target, distracter, and neutral stimuli of the two groups under (A) positive and (B) negative condition. Images in the top row show ERPs in the active group, and the bottom row shows the ERPs in the sham group. Both ERP waveforms of three types stimuli and averaged amplitude differences in N100, P200, and P300 components are shown. The shaded area in waveforms represent the time windows for each component. Tar pre = waveform elicited by target stimuli before stimulation; Tar post = waveform elicited by target stimuli after stimulation; Dis pre = waveform elicited by distracter stimuli before stimulation; Dis post = waveform elicited by distracter stimuli after stimulation; Neu pre = waveform elicited by neutral stimuli before stimulation; Neu post = waveform elicited by neutral stimuli after stimulation. (* represents the significant different, *p < 0.05; **p < 0.01; ***p < 0.005)

ERP waveforms of target, distracter and neutral stimuli were elicited and illustrated into four parts (two groups, two conditions, Fig. 3), with time window expression for each component. As depicted, the oddball paradigm exhibited typical visual ERP waveforms and retrieved three highly predictable ERP components of N100, P200 and P300. Considering the entrainment results, we focused on the ERP comparisons between IAF-tACS and sham group. First come to ANOVA results of ERP amplitude, for N100, we found a significant influence of the factor time (positive condition: F_1,46_ = 10.83, p = 0.002; negative condition: F_1,47_ =22.01, p = 0.000024), along with a significant effect of the factor condition (positive condition: F_2,92_ = 40.06, p = 3.06e-13; negative condition: F_1.7,81.9_ = 56.17, p = 8.94e-17). The factor group and all interactions were not significant (all p > 0.05 in two conditions), indicating an almost identical decrease in two groups and significant for most neutral and distracter stimuli in two conditions. One possible explanation to account for this finding was increased familiarity of the task after the first experiment. For P200, we found a significant influence of the factor time (positive condition: F_1,46_ = 6.00, p = 0.02; negative condition: F_1,45_ = 7.91, p = 0.007), along with a significant effect of the factor condition (positive condition: F_2,92_ = 12.30, p = 0.000037; negative condition: F_1.3,59.2_ = 8.908, p = 0.00029). The factor group was not significant (positive condition: F_1,46_ = 0.85, p = 0.36; negative condition: F_1,45_ = 0.49, p = 0.49). Besides, the crucial interaction of time × group was significant (positive condition: F_1,46_ = 6.38, p = 0.02; negative condition: F_1,45_ = 4.25, p = 0.04), but not significant for other interactions (all p > 0.05 in two conditions). A post hoc analysis revealed that after IAF-tACS, the P200 amplitude was significantly elevated in both positive (target: p = 0.009, distracter: p = 0.01, neutral: p = 0.03) and negative conditions (target: p = 0.01, distracter: p = 0.01, neutral: p = 0.02) but was not substantial in the sham group. This finding indicated a strong promotion of early attention of facial emotions after IAF-tACS compared to the sham group. For late ERP component P300, the ANOVA showed an only significant effect of condition (positive condition: F_2,88_ = 44.82, p = 3.79e-14; negative condition: F_1.7,77.7_ = 53.62, p = 6.28e-16), which was induced only by the target and distraction stimuli and with slight differences after stimulation between the two groups from post hoc analysis.

To verify the effectiveness of IAF-tACS on emotional attention, we conducted an additional ANOVA with P200 amplitude in 4 groups including IAF-tACS, 10 Hz tACS, sham and DC stimulation group. We found a significant influence of the factor group (positive condition: F_3,92_ = 7.43, p = 0.00016; negative condition: F_3,91_ = 5.45, p = 0.002), condition (positive condition: F_2,184_ = 23.49, p = 8.23e-10; negative condition: F_2,182_ = 8.53, p = 0.0003) and time for one condition (positive condition: F_1,92_ = 3.67, p = 0.59; negative condition: F_1,91_ = 9.59, p = 0.003), along with a significant effect of the interaction of time × group (positive condition: F_3,92_ = 2.74, p = 0.048; negative condition: F_3,91_ = 2.73, p = 0.048). Other interactions were not significant (all p > 0.05 in two conditions). A post hoc analysis revealed that after tACS, the P200 amplitude was significantly elevated by specific stimuli in both IAF (positive condition: p = 0.035 for target, p = 0.009 for distracter, p = 0.071 for neutral; negative condition: p = 0.016 for target, p = 0.05 for distracter, p = 0.03 for neutral) and 10 Hz-tACS groups (positive condition: p = 0.047 for target, p = 0.215 for distracter, p = 0.147 for neutral; negative condition: p = 0.031 for target, p = 0.01 for distracter, p = 0.02 for neutral), but was not substantial in sham or DC stimulation group. This finding indicated that active tACS modulated emotional attention compared to the sham group and the promotion was not induced by placebo effect. Besides, the P200 modulation by IAF-tACS exhibited a wider emotion range compared to 10 Hz group.

Regarding latency, we found no significant between-group effect of tACS for three components. there was a small decrease in both conditions for the active group, results were not observed in the sham group as well. Taken together, the results suggest the effective role of tACS on emotional attention, which was mainly embodied in P200 amplitude modulation rather than latency.

### Correlation analysis

**Fig. 4:**
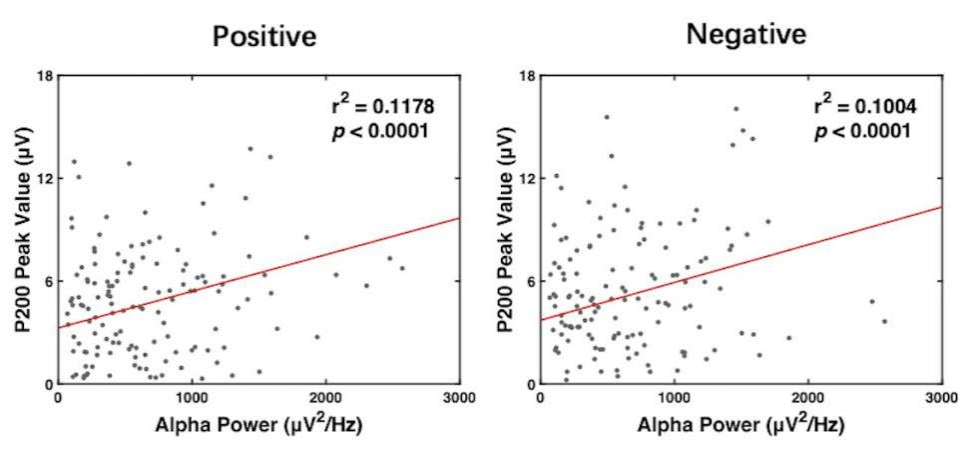
Linear Correlation Analysis. Linear correlation analysis between absolute individual alpha power (x-axis) and P200 peak value (y-axis) in positive (left) and negative (right) conditions. Each scatterplot represents one subject value from two sets of data. Regression lines are drawn in red lines. Significance of Pearson correlation (p) and R-square (r2) are marked in the upper left corners of the scatterplots

To explore a potential correlation between ERPs and at-rest neural oscillations, both correlation and regression analyses were conducted via Pearson correlations, which is known as a pervasive sample correlation coefficient (r^2^). Considering the main outcome of ERPs, P200 peak value, as well frontal alpha power pre- and post-tACS, from three tACS groups were integrated to create the scatter plot and measured linear relationships. As shown in Fig. 4, a significant positive correlation between P200 peak value and alpha power was found in both positive (r^2^ = 0.1178, p = 0.000022) and negative (r^2^ = 0.1004, p = 0.000097) conditions. Combining the above results, the enhanced emotional attention might be interpreted as alpha entrainment induced by IAF-tACS.

## Discussion

Overall, compared to the sham group, participants in the IAF-tACS group displayed increased alpha oscillatory patterns in the frontal lobe at rest, especially in the open state. Considering the origin of IAF, the alpha entrainment was more obvious than 10 Hz-tACS. ERP results revealed a unique increased P200 amplitude in two active groups after stimulation. However, the ERP modulation induced by IAF-tACS showed a much wider range of emotions compared to tACS at fixed frequency, indicating the advantage of individual stimulation pattern. Another finding was a significant positive correlation between frontal alpha power and P200 peak value. These results not only support the neuromodulatory role of the dlPFC in the emotional attention processing but also provide strong evidence that alpha-tACS can improve the capacity of emotion recognition at early stage by modulating alpha oscillations, which helps underpin the clinical potency of tACS in neuropsychiatric disorders related to emotion processing.

Based on previous evidence from ERP studies, an hypothesis suggesting that there are three stages of facial expression processing has been established(Luo et al. 2010). In the first stage, perceptual priming processing occurs with temporal characteristics of the N100 component. In this phase, familiarity can facilitate the automatic processing of facial information, which accounted for the significant decline in N100 amplitude in the two groups in this study(Goto and Tobimatsu 2005; Bornstein et al. 2013). Afterward, emotional information processing starts early attention to emotional stimuli within around 200 ms after stimuli onset. Distinct P200 has supported the association with initial attentional control, which has become a biomarker of attentional impairment for patients with depression(Hu et al. 2017).

An emotional vocalizations research suggested that priming effects were tagged by P200 for both familiar and unfamiliar voices, which excludes the influence of familiarity on P200 amplitude(Föcker et al. 2011). With these results, a significant elevation in P200 amplitude in response to distracted stimuli was found in both conditions, indicating that the improved response of emotion recognition for facial emotion had nothing to do with emotion types. For the third stage, there is emotional differentiation among various facial expressions and further evaluation related to corresponding affective valence. Based on the above findings, the results report remarkable effects of tACS on emotional attention modulation, instead of automatic or complex affective processing in the late period.

In our study, tACS was used for studying early attention modulation of emotion processing, we demonstrated that tACS may be a potential and feasible treatment in psychiatric population. The study also provides evidence that neuronal oscillations involved in specific neuropsychiatric disorders are associated with disturbances in other brain oscillatory, versus sensory stimulation. Compared to animal models, these physiological effects in healthy humans have greater possibilities for clinical translation because of the specificity of human neocortex, where endogenous ongoing activity in complex oscillations might either amplify or suppress the effects of alternating fields externally(Johnson et al. 2020).

Regarding resting performance, spatially-normalized alpha alterations in the open state were more sensitive to intervention, at which stimulation frequency originated. That means, externally applied based on intrinsic oscillation showed successful modulation of oscillatory network within target area, which clarifies the spatial and frequency specificity of IAF-tACS induced neural entrainment. Regrettably, the tACS effect on alpha power was not observed in 10 Hz group. We think both sample size, neuroanatomical and neurophysiological variability may account for that phenomenon, such as scalp thickness and scalp-to-cortex distance and alpha spectrum alternation(McConnell et al. 2001; Zanto et al. 2021). Interestingly, we found that tACS effect also depend on intrinsic oscillatory. Alpha activity was enhanced in participants with lower endogenous alpha activity, and subtle or even adverse alternations were found in participants with high endogenous alpha activity after stimulation. However, under conditions of higher endogenous alpha power, tACS failed to substantially affect alpha power in the close state and exhibited small increases after tACS (only in the active group). In line with a previous study, greater intensity is required to elevate alpha oscillations when a participant’s eyes are closed(Neuling et al. 2013).

In light of individual differences, tACS administered based on individual alpha activity revealed a more obvious elevation in individuals compared to the fixed alpha band. This finding is consistent with recent research on tACS, emphasizing its capability to entrain the activity of individual neurons in the primate brain (spatial- and frequency-specifically)(Krause et al. 2019). In both fixed and individual frequency bands, there was sharp alpha entrainment in the IAF group compared to 10 Hz tACS group under the same stimulation intensity. This phenomenon may be explained stimulation frequency selection of IAF, where corresponded to native regularity pattern of individual network. This finding provides a direct and robust evidence for parameter optimization of tACS.

Taking the positive correlation between resting alpha power and P200 value into account, it was found that the improved emotional attention may be explained by alpha entrainment via tACS. This finding relates individual brain oscillation at rest to brain activity during a task-related response. Taken together with the clinical avenue of P200 above, these novel findings verify the important role of resting EEG patterns in emotional research, which has also been proven by antidepressant response predictions in recent clinical tests(Wu et al. 2020).

Another innovative point in the study is dlPFC function. Recently, more and more studies have identified the key role of dlPFC in top-down cognitive-emotional control of the affective state, generating structures as well as process of attention responses(Notzon et al. 2018). For a long time, the dlPFC has been assumed to realize its executive function for affective states and subsequent behavior via dorsal system. For instance, pioneer studies showed hypoactivation of the right dlPFC during negative material processing in major depressive disorder (MDD)(Zhong et al. 2016). In addition, the dlPFC send signals to its adjacent regions such as the orbitofrontal cortex, dorsomedial prefrontal cortex, and hippocampus, to elicit an appropriate response(Ghashghaei and Barbas 2002; Kringelbach 2005; Rangel and Hare 2010). In situations where mood and emotion are involved, the dlPFC is always activated, especially during tasks that require direct attention. Notably, these findings have advanced the neurobiological understanding of the dlPFC’s function during emotional attention.

As mentioned previously, psychiatric disorders with aberrant emotional processes are likely to show some symptoms that are characteristic of lesions in the dlPFC(Phillips et al. 2003). For instance, previous studies indicated reduced cortical neuronal size and reduced glial cell density in schizophrenic patients, as well as reduced glial cell number and prefrontal cortex volumes in bipolar disorder patients(Ketter et al. 2001). Furthermore, studies in MDD patients pointed at the bilateral dlPFC hypoactivations during episode, mainly reflect in reduced density and dlPFC volumes along with reductions in metabolism and blood flow employing functional neuroimaging techniques(Rajkowska et al. 1999).

Except for these structural and functional abnormalities, dlPFC impairment is also reflected as abnormal neural oscillations, especially the alpha response. Recent research has explored alpha activity in the dlPFC activity (i.e., repetitive transcranial magnetic stimulation [rTMS], transcranial direct current stimulation [tDCS], and tACS)(Ironside et al. 2019).

Recent rTMS research demonstrated that excitatory rTMS induced in the dlPFC strengthened top-down control of aversive facial stimuli and induced effects on emotional arousal of fearful facial in MDD patients(Notzon et al. 2018). Thus, low-field magnetic stimulation synchronized to the IAF is thought to be more effective for MDD treatment(Leuchter et al. 2015). Additionally, anodal tDCS in the left dlPFC has resulted in obvious effects on emotion regulation. Another research study in our lab has discovered the emotional effects on alpha asymmetry, which has not yet been published. Thus far, few studies have tested the emotional or attentional effects of alpha-tACS in the dlPFC.

Another research recently found that 10 Hz-tACS significantly modulated emotional processing, and this study also discovered significant improvements in alpha entrainment and the P2 component of the emotional task after stimulation(Hu et al. 2021). However, a P3 enhancement was also find in this research. We did not find consistent result, may be due to variations in participant samples and statistical methods. When the results of our parameter optimization research were combined, the individual-specific strategy (IAF) outperformed fixed parameters in modulating emotional attention (10 Hz). However, there are some limitations in our research in terms of the indirect effect that may be caused by current shunting(Vöröslakos et al. 2018). Asamoah et al., for example, distinguished the transcranial and transcutaneous mechanisms of tACS by selectively blocking the pathways and found that tACS directly affect peripheral nerves while having an indirect effect on motor cortex activity(Asamoah et al. 2019). More effort is required for future research.

Although participants did not report any behavioral or psychological anomalies during the experiment, we cannot entirely exclude the differences in mood or other emotional characteristics between two groups, which would influence the results. However, most of the participants were university students, which could have weakened the influence of these factors.

Considering these limitations, the results demonstrated the entrainment of individual alpha-tACS and its essential role in emotional attention, at least in healthy subjects. However, it is not clear to what extent tACS can influence this function. Systematic comparison between other neurostimulation techniques for exact mechanism as well the combination of functional imaging techniques, which offer excellent resolution in both temporal and spatial domains, are still lacking.

The presented findings offer important insights into the crucial roles of prefrontal alpha measures and dlPFC function in the area of emotional attention. The revealing of such effects should encourage more research in order to explore the intrinsic mechanisms influencing the dlPFC and tACS as well their neurophysiologic correlates. With additional research, the neurophysiological profiles of emotion processing may serve as valuable biomarkers for diagnoses and reveal potential target brain regions for therapeutic interventions in various neuropsychiatric disorders.

## Data Availability

All data that support the findings of this study are available on request from the corresponding author.

## Appendix

### Detailed description of tACS setting

In the active group, total active stimulation required 20 min. In the sham group, only a short period of tACS was administered with 10 cycles ramp-up and 10 cycles ramp-down. This ensure that participants in the sham group experienced a slight acupuncture feeling, similar to the active group. However, participants in the sham group continued wearing the electrode cap after the stimulation phase to experience stimulation for the same duration. Stimulation frequency and intensity setting were the same in IAF-tACS groups.

### EEG Pre-processing

Preprocessing was performed offline using EEGLAB in the MATLAB (R2013b) environment, which is an open-source MATLAB-based toolbox for data processing of electrophysiological signals. The reference electrode was first adjusted to the binaural mean (M1 and M2) to avoid differences between the left and right hemispheres. Then, the raw resting EEG signals were down-sampled to 500 Hz and bandpass-filtered from 0.5 to 120 Hz, while down-sampled the task EEG signals to 200 Hz and bandpass-filtered from 0.5 to 45 Hz with a 50 Hz notch filter for both, then followed by independent component analysis (ICA) to remove artifacts caused by heartbeats and eye movements.

### ERP Acquisition

Raw EEG data was divided into three emotional states. Data was subjected to 1−30 Hz bandpass filtering to observe the waveform. All of the epochs were baseline-corrected using data from the first 200 ms pre-stimulus. Trials with amplitudes greater than 100µV were discarded by subtracting the minimum amplitude by maximum amplitude. Ultimately, averaging of dataset responses to three types of emotional stimuli resulted in three ERP waveforms for each participant (target, distracter, and neutral) within the cranial channels of interest.

## References

[CR1] Adolph D, Margraf J (2017). The differential relationship between trait anxiety, depression, and resting frontal α-asymmetry. J Neural Transm.

[CR2] Ahn S, Prim JH, Alexander ML, McCulloch KL, Fröhlich F (2019). Identifying and engaging neuronal oscillations by transcranial alternating current stimulation in patients with chronic low back pain: a randomized, crossover, double-blind, sham-controlled pilot study. J Pain.

[CR3] Alzueta E, Melcón M, Poch C, Capilla A (2019). Is your own face more than a highly familiar face?. Biol Psychol.

[CR4] Asamoah B, Khatoun A, Mc Laughlin M (2019). tACS motor system effects can be caused by transcutaneous stimulation of peripheral nerves. Nat Commun.

[CR5] Bardeen JR, Fergus TA, Orcutt HK (2012). An examination of the latent structure of the Difficulties in Emotion Regulation Scale. J Psychopathol Behav Assess.

[CR6] Barnes LL, Harp D, Jung WS (2002). Reliability generalization of scores on the Spielberger state-trait anxiety inventory. Educ Psychol Meas.

[CR7] Bazanova O, Vernon D (2014). Interpreting EEG alpha activity. Neurosci Biobehavioral Reviews.

[CR8] Bocchio M, Nabavi S, Capogna M (2017). Synaptic plasticity, engrams, and network oscillations in amygdala circuits for storage and retrieval of emotional memories. Neuron.

[CR9] Bornstein MH, Arterberry ME, Mash C (2013). Differentiated brain activity in response to faces of “own” versus “unfamiliar” babies in primipara mothers: An electrophysiological study. Dev Neuropsychol.

[CR10] Campanella S, Gaspard C, Debatisse D, Bruyer R, Crommelinck M, Guerit J-M (2002). Discrimination of emotional facial expressions in a visual oddball task: an ERP study. Biol Psychol.

[CR11] Connell AM, Danzo S, Magee K, Uhlman R (2019). Children’s appraisals of maternal depression and responses to emotional faces in early-adolescence: An Event Related Potential (ERP) study. J Affect Disord.

[CR12] Crost NW, Pauls CA, Wacker J (2008). Defensiveness and anxiety predict frontal EEG asymmetry only in specific situational contexts. Biol Psychol.

[CR13] Dixon ML, Thiruchselvam R, Todd R, Christoff K (2017). Emotion and the prefrontal cortex: an integrative review. Psychol Bull.

[CR14] Dolcos F, Katsumi Y, Moore M, Berggren N, de Gelder B, Derakshan N, Hamm AO, Koster EH, Ladouceur CD, Okon-Singer H (2020). Neural correlates of emotion-attention interactions: From perception, learning, and memory to social cognition, individual differences, and training interventions. Neurosci Biobehavioral Reviews.

[CR15] Eidelman-Rothman M, Levy J, Feldman R (2016). Alpha oscillations and their impairment in affective and post-traumatic stress disorders. Neurosci Biobehavioral Reviews.

[CR16] Fields EC, Kuperberg GR (2012). It’s all about you: An ERP study of emotion and self-relevance in discourse. NeuroImage.

[CR17] Föcker J, Hölig C, Best A, Röder B (2011). Crossmodal interaction of facial and vocal person identity information: An event-related potential study. Brain Res.

[CR18] Ghashghaei H, Barbas H (2002). Pathways for emotion: interactions of prefrontal and anterior temporal pathways in the amygdala of the rhesus monkey. Neuroscience.

[CR19] Goto Y, Tobimatsu S (2005) An electrophysiological study of the initial step of face perception. In International Congress Series. Elsevier, pp 45–48

[CR20] Hanslmayr S, Axmacher N, Inman CS (2019). Modulating human memory via entrainment of brain oscillations. Trends Neurosci.

[CR21] Harmon-Jones E, Abramson LY, Nusslock R, Sigelman JD, Urosevic S, Turonie LD, Alloy LB, Fearn M (2008). Effect of bipolar disorder on left frontal cortical responses to goals differing in valence and task difficulty. Biol Psychiatry.

[CR22] Hu B, Rao J, Li X, Cao T, Li J, Majoe D, Gutknecht J (2017). Emotion regulating attentional control abnormalities in major depressive disorder: an event-related potential study. Sci Rep.

[CR23] Hu P, He Y, Liu X, Ren Z, Liu S (2021) Modulating emotion processing using transcranial alternating current stimulation (tACS)-A sham-controlled study in healthy human participants. In: 2021 43rd Annual International Conference of the IEEE Engineering in Medicine & Biology Society (EMBC), pp 6667–6670: IEEE 10.1109/EMBC46164.2021.9630564 34892637

[CR24] Imperatori C, Farina B, Quintiliani MI, Onofri A, Gattinara PC, Lepore M, Gnoni V, Mazzucchi E, Contardi A, Della Marca G (2014). Aberrant EEG functional connectivity and EEG power spectra in resting state post-traumatic stress disorder: A sLORETA study. Biol Psychol.

[CR25] Ironside M, Browning M, Ansari TL, Harvey CJ, Sekyi-Djan MN, Bishop SJ, Harmer CJ, O’Shea J (2019). Effect of prefrontal cortex stimulation on regulation of amygdala response to threat in individuals with trait anxiety: a randomized clinical trial. JAMA psychiatry.

[CR26] Jacobs J, Kahana MJ, Ekstrom AD, Fried I (2007). Brain oscillations control timing of single-neuron activity in humans. J Neurosci.

[CR27] Jessen S, Grossmann T (2020). The developmental origins of subliminal face processing. Neurosci Biobehavioral Reviews.

[CR28] Johnson L, Alekseichuk I, Krieg J, Doyle A, Yu Y, Vitek J, Johnson M, Opitz A (2020). Dose-dependent effects of transcranial alternating current stimulation on spike timing in awake nonhuman primates. Sci Adv.

[CR29] Junghöfer M, Bradley MM, Elbert TR, Lang PJ (2001) Fleeting images: a new look at early emotion discrimination. In: Wiley Online Library 11347862

[CR30] Kanai R, Chaieb L, Antal A, Walsh V, Paulus W (2008). Frequency-dependent electrical stimulation of the visual cortex. Curr Biol.

[CR31] Kang SS, MacDonald AW, Chafee MV, Im C-H, Bernat EM, Davenport ND, Sponheim SRJCN (2018). Abnormal cortical neural synchrony during working memory in schizophrenia. Clin Neurophysiol.

[CR32] Ketter TA, Kimbrell TA, George MS, Dunn RT, Speer AM, Benson BE, Willis MW, Danielson A, Frye MA, Herscovitch P, Post RM (2001). Effects of mood and subtype on cerebral glucose metabolism in treatment-resistant bipolar disorder. Biol Psychiatry.

[CR33] Krause MR, Vieira PG, Csorba BA, Pilly PK, Pack CC (2019) Transcranial alternating current stimulation entrains single-neuron activity in the primate brain. Proceedings of the National Academy of Sciences 116:5747–5755 10.1073/pnas.1815958116 PMC6431188 30833389

[CR34] Kringelbach ML (2005). The human orbitofrontal cortex: Linking reward to hedonic experience. Nat Rev Neurosci.

[CR35] Leuchter AF, Cook IA, Feifel D, Goethe JW, Husain M, Carpenter LL, Thase ME, Krystal AD, Philip NS, Bhati MT (2015). Efficacy and safety of low-field synchronized transcranial magnetic stimulation (sTMS) for treatment of major depression. Brain Stimul.

[CR36] Liston C, Chen AC, Zebley BD, Drysdale AT, Gordon R, Leuchter B, Voss HU, Casey B, Etkin A, Dubin MJ (2014). Default mode network mechanisms of transcranial magnetic stimulation in depression. Biol Psychiatry.

[CR37] Luo W, Feng W, He W, Wang N-Y, Luo Y-J (2010). Three stages of facial expression processing: ERP study with rapid serial visual presentation. NeuroImage.

[CR38] Martínez A, Tobe R, Dias EC, Ardekani BA, Veenstra-VanderWeele J, Patel G, Breland M, Lieval A, Silipo G, Javitt DC (2019). Differential patterns of visual sensory alteration underlying face emotion recognition impairment and motion perception deficits in schizophrenia and autism spectrum disorder. Biol Psychiatry.

[CR39] McConnell KA, Nahas Z, Shastri A, Lorberbaum JP, Kozel FA, Bohning DE, George MS (2001). The transcranial magnetic stimulation motor threshold depends on the distance from coil to underlying cortex: a replication in healthy adults comparing two methods of assessing the distance to cortex. Biol Psychiatry.

[CR40] Neuling T, Rach S, Herrmann CS (2013). Orchestrating neuronal networks: sustained after-effects of transcranial alternating current stimulation depend upon brain states. Front Hum Neurosci.

[CR41] Notzon S, Steinberg C, Zwanzger P, Junghofer M (2018). Modulating Emotion Perception: Opposing Effects of Inhibitory and Excitatory Prefrontal Cortex Stimulation. Biol psychiatry Cogn Neurosci neuroimaging.

[CR42] Phillips ML, Drevets WC, Rauch SL, Lane RJBp (2003). Neurobiology of emotion perception I: The neural basis of normal emotion perception. Biol Psychiatry.

[CR43] Rajkowska G, Miguel-Hidalgo JJ, Wei J, Dilley G, Pittman SD, Meltzer HY, Overholser JC, Roth BL, Stockmeier CAJBp (1999). Morphometric evidence for neuronal and glial prefrontal cell pathology in major depression. Biol Psychiatry.

[CR44] Rangel A, Hare T (2010). Neural computations associated with goal-directed choice. Curr Opin Neurobiol.

[CR45] Raschle NM, Fehlbaum LV, Menks WM, Martinelli A, Prätzlich M, Bernhard A, Ackermann K, Freitag C, De Brito S, Fairchild G (2019) Atypical Dorsolateral Prefrontal Activity in Female Adolescents With Conduct Disorder During Effortful Emotion Regulation. Biological Psychiatry: Cognitive Neuroscience Neuroimaging 4:984–994 10.1016/j.bpsc.2019.05.003 PMC6838678 31311717

[CR46] Sohal VS (2012). Insights into cortical oscillations arising from optogenetic studies. Biol Psychiatry.

[CR47] Vöröslakos M, Takeuchi Y, Brinyiczki K, Zombori T, Oliva A, Fernández-Ruiz A, Kozák G, Kincses ZT, Iványi B, Buzsáki G (2018). Direct effects of transcranial electric stimulation on brain circuits in rats and humans. Nat Commun.

[CR48] Wu W, Zhang Y, Jiang J, Lucas MV, Fonzo GA, Rolle CE, Cooper C, Chin-Fatt C, Krepel N, Cornelssen CA (2020). An electroencephalographic signature predicts antidepressant response in major depression. Nat Biotechnol.

[CR49] Yavari F, Jamil A, Samani MM, Vidor LP, Nitsche MA (2018). Basic and functional effects of transcranial Electrical Stimulation (tES)—An introduction. Neurosci Biobehavioral Reviews.

[CR50] Zanto TP, Jones KT, Ostrand AE, Hsu W-Y, Campusano R, Gazzaley A (2021). Individual differences in neuroanatomy and neurophysiology predict effects of transcranial alternating current stimulation. Brain Stimul.

[CR51] Zheng J, Stevenson RF, Mander BA, Mnatsakanyan L, Hsu FP, Vadera S, Knight RT, Yassa MA, Lin JJ (2019). Multiplexing of theta and alpha rhythms in the amygdala-hippocampal circuit supports pattern separation of emotional information. Neuron.

[CR52] Zhong X, Pu W, Yao S (2016). Functional alterations of fronto-limbic circuit and default mode network systems in first-episode, drug-naïve patients with major depressive disorder: a meta-analysis of resting-state fMRI data. J Affect Disord.

